# Proton motive force generated by microbial rhodopsin promotes extracellular electron transfer

**DOI:** 10.1016/j.synbio.2025.01.001

**Published:** 2025-01-07

**Authors:** Wenqi Ding, Tong Lin, Yun Yang, Wen-Wei Li, Shaoan Cheng, Hao Song

**Affiliations:** a Frontiers Science Centre for Synthetic Biology (Ministry of Education), And Key Laboratory of Systems Bioengineering, School of Chemical Engineering and Technology, Tianjin University, Tianjin, 300072, China; b College of Life Science, Langfang Normal University, Langfang, Hebei, 065000, China; c Beijing Advanced Innovation Centre for Biomedical Engineering, Key Laboratory for Biomechanics and Mechanobiology of Ministry of Education, School of Engineering Medicine, Beihang University, Beijing, 100083, China; d Chinese Academy of Sciences Key Laboratory of Urban Pollutant Conversion, Department of Environmental Science and Engineering, University of Science & Technology of China, Hefei, 230026, China; e State Key Laboratory of Clean Energy, Department of Energy Engineering, Zhejiang University, Hangzhou, 310027, China; f Haihe Laboratory of Sustainable Chemical Transformations, Tianjin, 300192, China

**Keywords:** Extracellular electron transfer, Microbial rhodopsin, Proton motive force, *Shewanella oneidensis* MR-1

## Abstract

The primary limitation to the practicability of electroactive microorganisms in bioelectrochemical systems lies in their low extracellular electron transfer (EET) efficiency. The proton motive force (PMF) represents the electrochemical gradient of protons generated by electron transport and proton pumping across the cytoplasmic membrane, serving as a crucial energy transfer pathway in bacterial membranes. Nevertheless, the impact of PMF on the EET efficiency remains ambiguous, while the microbial rhodopsin offers a simple and efficient avenue for non-photosynthetic cells to harness PMF. Here, we studied the function of three microbial rhodopsins (Arch, Mac, and cR-1) in facilitating EET via their heterologous expression in *S. oneidensis*, a model electroactive microorganism. Among these, the recombinant strain expressing rhodopsin cR-1 exhibited the highest output power density of 0.87 W/m^2^, 3.49-fold increase over the wild-type *S. oneidensis* MR-1. Our further transcriptomics analyses of the energy and materials metabolism of strain cR-1 showed that the underlying mechanism of enhanced EET efficiency was resulted from heterologous expression of the light-driven proton pump. The results suggested that strain cR-1 effectively expels protons to generate additional PMF and provide extra ATP supply to the cells, which facilitated lactate uptake and utilization, thus enhancing electrons generation in cells. This augmented intracellular electron pool capacity ultimately resulted in enhancement of EET rate and power generation efficiency of the recombinant *S. oneidensis*.

## 1 Introduction

Microbial bioelectrochemical systems (BESs) utilize materials metabolism and extracellular electron transfer (EET) process of electroactive microorganisms to convert chemical energy into electrical energy, which enables a diverse array of applications [[1], [2], [3], [4]], including microbial fuel cells (MFCs) for bioelectricity production in wastewater treatment [1], microbial electrolysis cells for hydrogen production [1,2], microbial desalination cells for seawater desalination [3], and synthesis of essential chemical compounds via microbial electrosynthesis [5,6]. In the past decades, extensive research efforts have been devoted to elucidating the molecular mechanisms underlying EET [7] and enhancing its efficiency through diverse approaches, encompassing synthetic biology modifications [8]advanced electrode materials [9], innovative bio-electrochemical reactor design [9]. However, the limited EET efficiency remains to be a major obstacle that impedes wide adoption of BES in industrial applications.

*Shewanella oneidensis* has emerged as a valuable model for studying EET due to its well-defined genetic information and relatively straightforward genetic manipulation. Over the past two decades, extensive and in-depth investigations have been conducted by numerous researchers to explore the fundamental aspects and technological significance of its EET [1,5]. These studies primarily focused on elucidating electron transfer components [10], enhancing electron shuttles yield [11], and developing three-dimensional hybrid biofilms through material engineering [12]. Most of these studies on enhancing EET efficiency have primarily focused on facilitating EET between bacterial and electrodes. Nevertheless, it remains elusive the impact of intracellular energy generation on the EET efficiency.

Living microorganisms obtain energy by controlling the flow of electrons through respiration, which involves complex redox component networks from electron donors to acceptors [13]. The electron transfer contributes to the generation of ion gradients (such as proton motive force) across membranes composed of chemical components (Δ[ions]) and electrical components (membrane potential, Δψ), providing energy for various cellular functions [14]. An electrochemical proton gradient across the cytoplasmic membrane, known as the proton motive force (PMF), drives essential cellular processes. For example, PMF drives ATP synthesis [15], enables transport of a diverse range of substrates including essential ions and metabolites [16,17], and motility [18]. Moreover, PMF plays a pivotal role in cell division [19] and cell-to-cell signaling [20].

Classic experiments in microbial bioenergetics have utilized light-driven reactions from bacteriorhodopsin or photosynthetic reaction centers as temporary driving forces for understanding transport and chemosmosis [[21], [22], [23]]. Bacteriorhodopsin is one of the simplest mechanisms that harnesses isomerization of retinal to achieve light-driven proton translocation facilitating substance transport and energy production [24]. The light-dependent membrane potential generated by bacteriorhodopsin in membrane vesicles isolated from a native host serves as the driving force for ATP synthesis [23] and the uptake of leucine and glutamate [25,26]. Bacteria rhodopsins have been shown to promote growth [27], enhance flagella motility in *E. coli* [28], and facilitate hydrogen production [29], highlighting their potential as versatile tools for biotechnological applications.

Based on these fundamental studies, three distinct light-driven proton pumps, which provided a straightforward and controllable source of proton motive force for ATP generation, were individually overexpressed in the recombinant *S. oneidensis* strains, Arch-3, Mac, cR-1, respectively. Among these strains, strain cR-1 exhibited the highest EET efficiency, which was primarily manifested through a significant increase in power generation output in MFCs. Additionally, strain cR-1 demonstrated enhanced ATP synthesis, accelerated lactate consumption, increased acetate production, and elevated levels of NADH and NAD^+^ in comparison to the control strain (*i.e.*, the wild-type *S. oneidensis* MR-1). By utilizing the whole-genome gene expression profiling analysis, we investigated the physiological responses and the underlying molecular mechanisms of enhanced EET efficiency of *S. oneidensis* induced by the light-driven proton pump. Our research findings suggest that the proton motive force generated by bacteria rhodopsin photophosphorylation serves as a crucial energy source for both intracellular and extracellular electron transfer processes in *S. oneidensis*, which holds paramount significance in enhancing the EET efficiency.

## 2 Methods and materials

### 2.1 Gene synthesis, plasmid construction and bacterial culture

The three microbial rhodopsins used in this study are *Halorubrum sodomense* archaerhodopsin-3 (Arch/aR-3) gene *aop3* [30,31], *Leptosphaeria maculans* opsin (Mac/LR/Ops) gene *ops* [30,32] and *Haloarcula argentinensis* cruxrhodopsin-1 (cR-1) gene *cop-1* [30,33]. The gene coding sequence were extracted from NCBI and adapted for expression in *S. oneidensis* MR-1 using a java codon optimization tool (http://www.jcat.de/) to mitigate translation impediments caused by limited availability of tRNAs for rare codons. Each gene component was synthesized as a biobrick, and the codon-optimized sequences were designed to exclude restriction enzyme sites of *Eco*RI, *Xba*I, *Spe*I, and *Pst*I. The optimized gene sequence was flanked by an upstream prefix (containing *Eco*RI and *Xba*I), a ribosome binding site (BBa_B0034, iGEM), and a downstream suffix (containing *Spe*I and *Pst*I). The designed gene sequences were synthesized in vitro and validated through Sanger sequencing analysis.

### 2.2 Recombinant strain construction and strain cultivation

*E. coli* DH5α was utilized for all molecular biology procedures, while plasmids were transferred to *S. oneidensis* MR-1 through conjugation with the mating strain *E. coli* WM3064. 100 μg/mL of 2,6-diaminopimelic acid (DPA) was supplemented to support the growth of *E. coli* WM3064. Subsequently, for plasmid maintenance, kanamycin was added to the culture medium at a concentration of 50 μg/mL when necessary. To facilitate multigene assembly in *S. oneidensis*, we employed a Biobrick-compatible expression vector pYYDT, previously constructed in our laboratory, which allows for differential IPTG induction and enables various levels of gene expression. The *S. oneidensis* MR-1 strains harboring plasmid pYYDT, pYYDT -*aop3*, pYYDT-*ops*, pYYDT-*cop-1* were named as strain MY, strain Arch, strain Mac, strain cR-1, respectively.

### 2.3 MFC setup

For the current production studies, cells were cultivated in a two-chamber microbial fuel cell (MFC) with chamber volumes of 140 mL. In this setup, a carbon cloth anode measuring 1.0 cm × 1.0 cm was separated from a carbon cloth cathode measuring 2.5 cm × 3.0 cm by employing a Nafion 117 membrane. Prior to inoculation, the system was subjected to a 1 h treatment with 3 % hydrogen peroxide at 80 °C, followed by thorough rinsing with distilled water washing with 0.5 M sulfuric acid. Finally, sterile distilled water was used for further rinsing. The cathodic electrolyte consisted of a 50 mM K_3_[Fe (CN)_6_], 50 mM KH_2_PO_4_, and 50 mM K_2_HPO_4_. The anode and cathode were connected via a 2 kΩ resistor, while the microbial fuel cells were incubated at a temperature of 30 °C. Output voltages were continuously recorded using MPS110001 data acquisition cards.

For current production studies, colonies of *S. oneidensis* MR-1were cultured in LB medium overnight. Subsequently, a 1 % inoculum was transferred into fresh LB broth supplemented with 50 μg/mL kanamycin and different concentrations of isopropyl-β-*d*-thiogalactoside (IPTG). After incubation for 12 h at 30 °C with 200 rpm, the cell suspension was evenly distributed into the anode chambers of three H-type MFCs at a final concentration of OD_600_ = 0.8. The anolyte in these MFCs consisted of M9 buffer, mineral-vitamin mixture [34], 2.5 g/L casamino acid, 1 mM MgSO_4_, 0.1 mM CaCl_2_, 18 mM lactate, 50 μg/mL kanamycin, and different concentrations of IPTG. Prior to introduction of the cell suspension, N_2_ gas was passed through the anode chambers in order to remove any traces of oxygen.

Chronoamperometry of the strains interacting with a carbon cloth working electrode poised at +0.2 V *vs.* Ag/AgCl (KCl saturated) reference electrode. The lighting condition was provided by an underground lamp with a power rating of 9 W, emitting light at a central wavelength of 550 ± 30 nm. The lamp was positioned 2 cm away from the anode chamber under lighting conditions. The illuminance-meter recorded an average luminance of approximately 10,000 lux. Linear sweep voltammetry (LSV) by gradually decreasing the potential from the open circuit potential (OCP) to −0.3 V at a scan rate of 0.1 mV/s, utilizing a CHI1000C electrochemical workstations (CH Instrument, Shanghai, China).

### 2.4 Light-driven proton pump absorption spectroscopy

Overnight *S. oneidensis* (the recombinant strains and MY) suspensions were inoculated into LB broth (at a ratio of 1:100) supplemented with 50 μg/mL kanamycin and different concentrations of IPTG. The strains were cultured and induced for expression in LB medium for 12 h. After sampling, the cells were centrifuged and resuspended in 50 mM Tris-HCl (pH 8.0), 150 mM NaCl. Subsequently, the cells were disrupted under ice bath ultrasonication conditions, followed by the supernatant was taken through centrifugation (4 °C, 6000 rpm, 20 min). Finally, the pellet was resuspended using 150 mM NaCl solution after discarding the supernatant through high-speed centrifugation (4 °C, 12,000 rpm, 90 min). The above suspension should be transferred into a 96-well plate and added *all-trans* retinal (*at*RAL) to achieve a final concentration of 10 μM/L. Subsequently, the absorbance spectrum should be measured at wavelengths ranging from 450 nm to 650 nm at both 0 and 30 min. The absorption changes at each wavelength were normalized by subtracting the baseline data at 0 min.

### 2.5 Proton pump experiments

The strains retrieved and activated overnight in LB medium supplemented with 50 μg/mL kanamycin. The strains were then inoculated 50 μg/mL kanamycin and cultured at 30 °C with agitation at 200 rpm for 12 h. For strains Arch, Mac and cR-1, induction was achieved by adding 10 μM, 10 μM, and 20 μM IPTG to the LB medium, respectively. The bacterial cells were collected by centrifugation at 5000 rpm for 10 min the cells were resuspended separately in 10 mM NaCl, 10 mM MgSO_4_, and 100 μM CaCl_2_. This washing procedure was repeated 2–3 times. The bacterial cells were resuspended in a PBS buffer solution at a mass/volume ratio of 1:10. The resuspended cells were ultimately supplemented without or with *at*RAL to achieve a final concentration of 10 μM, followed by subsequent incubation in darkness. The pH meter (METTLER TOLEDO) was immersed into the resuspended solution until the pH stabilized. The initial pH value of the solution was recorded as 0, and a dark-light-dark cycle was performed with each stage lasting for 10 min. The temporal variation in pH was continuously monitored at intervals of every minute or every 2 min. After a specific duration of illumination, the protonophore carbonyl cyanide *m*-chlorophenyl hydrazone (CCCP) was promptly introduced until reaching a final concentration of 20 μM to investigate the impact of CCCP addition on pH in strains subsequent to light exposure.

### 2.6 Membrane potential measurement

A flow cytometry-based assay was applied to measure the PMF/membrane potential by using the fluorescence probe 3,3′-Diethyloxacarbocyanine Iodide DiOC_2_(3). The strains cR-1 and MY grown and induced expression of proton pump aerobically as described before. Following 12 h incubation in the absence of light, the cells were harvested via centrifugation. Subsequently, the samples were washed sequentially with 10 mM NaCl, 10 mM MgSO_4_, and 100 μM CaCl_2_ solutions. The bacterial cells were then collected by centrifugation and finally resuspended in PBS buffer. *at*RAL was added to achieve a final concentration of 10 μM, followed by exposure to LED light for a duration of 10 min and cells (approximately at a cell density of 10^6^ cells/mL) were extracted for flow cytometric analysis. To depolarize the membrane potential, the protonophore CCCP was added to a final concentration of 20 μM.

### 2.7 Lactate metabolism assay

To quantify the concentrations of metabolites in the medium obtained from electrochemical chambers, samples were extracted from the chambers, subjected to centrifugation. Lactate and acetate in the anolytes were determined by employing high-performance liquid chromatography (HPLC) with an organic acid column (Aminex Column, 300 mm × 7.8 mm, Bio-Rad), which underwent incubation at a temperature of 65 °C utilized a refractive index detector from Shimadzu Corporation (RID-20 A). The mobile phase comprised of 5 mM H_2_SO_4_ flowing at a rate of 0.6 mL min^−1^. The standard curves were established by employing known concentrations of each chemical, and the peak areas were integrated using the Agilent ChemStation software.

### 2.8 ATP measurement

The strains MY and cR-1 were cultured overnight in LB medium. Subsequently, 1 % of the inoculum was transferred to LB broth supplemented with 50 μg/mL kanamycin (strain cR-1 received an additional 20 μM IPTG). Following 12 h incubation, the bacterial suspensions were introduced into the anode chamber containing the aforementioned anolyte (strain cR-1 group also received IPTG at a final concentration of 20 μΜ, while both strains were treated with 2.5 μΜ *at*RAL). OD_600_ was adjusted to a final value of 0.8 for both strains MY and cR-1, and nitrogen gas was introduced into the anode chamber to eliminate any residual oxygen traces. The anode chamber was placed on the magnetic stirrer at 30 °C with 50 rpm under dark or light conditions. Once the voltage output reached its peak, acetic acid was rapidly added to the anode chamber achieving a final concentration of 5 g/L, followed by continued cultivation under dark or light for another 4 h.

Samples of the liquid culture before and after treatment with acetic acid were taken, and their OD_600_ values were measured and adjusted to be consistent. Pellet the cells by centrifugation at 5000 rpm for 10 min. Resuspend the resulting pellet in a buffer solution containing 50 mM Tris-HCl at pH 8.0. Perform ice bath sonication using CV 334 ultrasonic disruptor (Scientz, China). Centrifuge once again at 4 °C and 12,000 rpm for 5 min to collect the supernatant. The reagents and samples were added to a 96-well plate, as instructed by the ATP content assay kit (Solarbio). Subsequently, the absorption intensity of the characteristic peak at 340 nm was measured using the microplate reader (SpectraMax M2/M2e/M3/M4/M5/M5e, Molecular Devices, USA). Finally, the ATP concentration was determined utilizing the calculation method provided in the kit. Three biological replicates were performed for each condition.

### 2.9 Quantification of intracellular NADH and NAD^+^

Once the output voltage reached its peak, carefully detached the carbon cloth electrode and promptly submerged it in an ice bath to inhibit cellular metabolism. Subsequently, retrieved the cells through centrifugation (4 °C, 12,000 rpm, 2 min). The total amount of NAD^+^ and NADH, as well as the individual quantity of NADH, were determined following the protocol provided by the NAD^+^/NADH Assay Kit with WST-8 (Beyotime). Subsequently, the amount of NAD^+^ was calculated based on the results obtained from the initial two steps.

### 2.10 Transcriptome analysis

Cells were harvested from the carbon cloth electrode in MFCs through rapid centrifugation at 12,000 rpm for 2 min upon reaching their peak output voltage, followed by immediate freezing in liquid nitrogen. The total RNA was extracted using the RNAprep pure Cell/Bacteria Kit (Tiangen) following lysozyme treatment. RNA degradation and contamination were monitored on 1 % agarose gels. The integrity and concentration of RNA were assessed using the RNA Nano 6000 Assay Kit of the Bioanalyzer 2100 system (Agilent Technologies, CA, USA). mRNA was purified from total RNA using probes to remove rRNA. Identical RNA was used as an input per sample. Sequencing libraries were generated using a NEBNext®UltraTM RNA Library Prep Kit for Illumina® (NEB, USA) following the manufacturer's instructions, and index codes were added to attribute sequences to each sample. The clustering of the index-coded samples was performed on a cBot Cluster Generation System using TruSeq PE Cluster Kit v3-cBot-HS (Illumina) according to the manufacturer's instructions. After cluster generation, the library preparations were sequenced on an Illumina Novaseq platform and paired-end reads were generated. The data were analyzed by Beijing Novogene Bioinformatics Technology Co., Ltd. (China). MY and cR-1 represent control and experimental group, respectively.

## 3 Results and discussion

### 3.1 Expression of microbial rhodopsin in recombinant *S. oneidensis* enhanced power generation

**Fig. 1:**
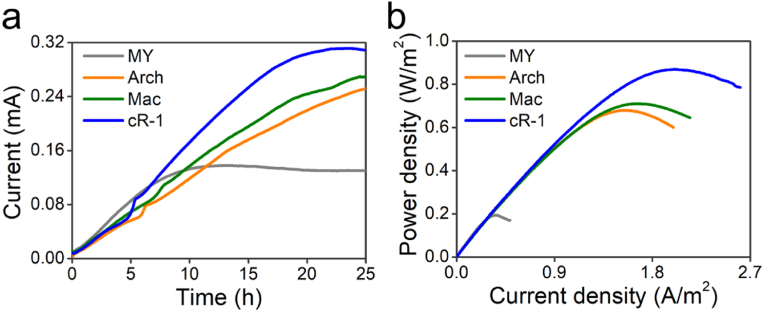
Electrochemical analysis of the strain MY (bearing an empty plasmid) and three recombinant strains Arch, Mac, cR-1. (a) Chronoamperometry of the strains MY, Arch, Mac, and cR-1 interacting with working electrode (carbon cloth) poised at 0.2 V *vs.* Ag/AgCl. (b) Power density output curves of the strains MY, Arch, Mac, and cR-1. Data are the average of three replicates for each strain.

The microbial rhodopsin genes *aop3*, *ops*, *cop-1* were expressed in *Shewanella oneidensis* MR-1 under the control of the inducible promoter P_tac_ (Fig. S1a). In microbial fuel cells (MFCs), optimization of IPTG and *at*RAL concentrations is imperative to maximize power generation performance in the recombinant *Shewanella* strains expressing microbial rhodopsins under light condition, as depicted in Fig. S2. Cultured wild-type empty plasmid strains MY and the recombinant strains cR-1 were introduced into the MFC anode chamber equipped with a working electrode poised at +0.2 V (*vs.* Ag/AgCl). The current versus time curves obtained during the electrochemical cultivation of these strains under light condition was presented in Fig. 1a. Compared to the strain MY, the recombinant strains Arch, Mac, and cR-1 exhibited significantly enhanced current output. The current outputs of the recombinant strains Arch, Mac, and cR-1 were 0.83-fold, 0.96-fold, and 1.26-fold higher than that of the strain MY, respectively, with corresponding currents of 0.25 mA, 0.27 mA, and 0.31 mA. The recombinant strains Arch, Mac, and cR-1 exhibited an augmented light-dependent current output or electron transfer capacity, whereas the strain MY did not demonstrate such increase in current output. While the recombinant strains Arch, Mac, and cR-1 exhibited a similar current output to that of strain MY under dark condition, as shown in Fig. S4a.These observations demonstrated that *S. oneidensis* expressing light-driven proton pumps can effectively harness light energy to enhance electric current generation.

Further electrochemical analysis was conducted to investigate the performance of these engineered strains. The MFC performance was assessed by conducting the linear sweep voltammetry (LSV) at a low scan rate of 0.1 mV/s from its open circuit potential (OCP). The polarization curves (Fig. S1b) reveal that the recombinant strains Arch, Mac, and cR-1 exhibit a significantly diminished slope compared to the strain MY, indicating a notable decrease in internal resistance in the MFCs. The output performance of the current density-power density relationship in the recombinant *Shewanella* strains under light condition was depicted in Fig. 1b. In comparison to the strain MY, the strains Arch, Mac, and cR-1 expressing microbial rhodopsin exhibited a significant enhancement in power output by 2.51-, 2.67-, and 3.49-fold, respectively, resulting in output power density of 0.67 ± 0.04 W/m^2^, 0.71 ± 0.03 W/m^2^, and 0.87 ± 0.03 W/m^2^, respectively. While under darkness condition, the recombinant strains Arch, Mac, and cR-1 demonstrated a comparable output power density to that of strain MY, as depicted in Fig. S4b. These results further substantiate that the exogenous proton pumps enhance the EET efficiency of the recombinant *Shewanella* strains. Under light condition, the expression of microbial rhodopsins significantly enhanced EET efficiency in MFCs, with the strain cR-1 demonstrated the most pronounced enhancement in power generation. Consequently, follow-up investigations would predominantly concentrate on the strain cR-1 to elucidate the mechanism underlying the augmentation of EET facilitated by the light-driven proton pump.

### 3.2 Characterization of recombinant proton pump in *S. oneidensis*

#### 3.2.1 Absorption spectrum detection

**Fig. 2:**
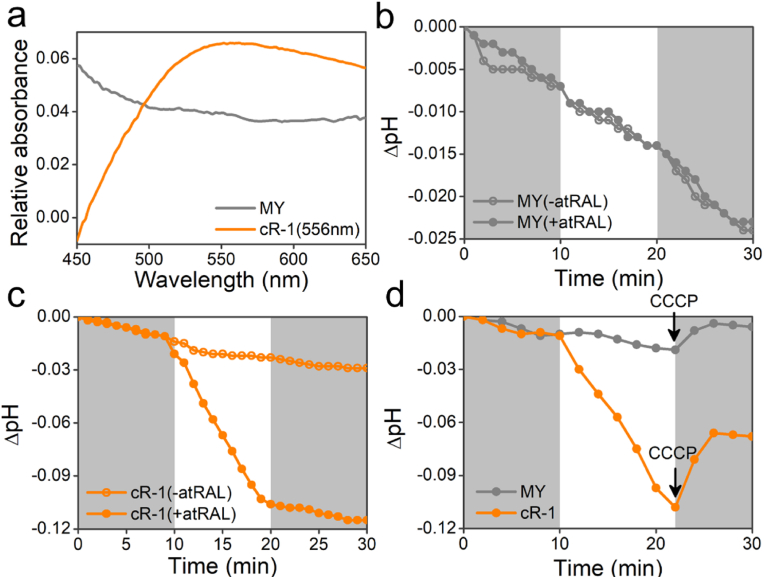
Absorption spectrum and validation of the light-driven proton pumping activity of wild-type empty plasmid strain MY and recombinant strain cR-1. Darkness condition (0–10 min and 20–30 min) are represented by gray shade, while the light condition (10–20 min) is represented by white shade. (a) Absorption spectrum of strain MY and strain cR-1. Relative absorbance is the absorbance change of crude membrane after adding *at*RAL. (b) The pH changes of strain MY were observed under a dark-light-dark cycle, both in the absence (gray hollow: *at*RAL) and presence (gray solid: +*at*RAL) of *at*RAL. (c) The pH changes of strain cR-1were observed under a dark-light-dark cycle, both in the absence (orange hollow: *at*RAL) and presence (orange solid: +*at*RAL) of *at*RAL. (d) The pH changes of strains MY and cR-1 were observed after addition of 20 μM CCCP following 10 min of light exposure. The black arrow represents the addition of CCCP (20 μM).

To characterize the properties and functions of the recombinant proton pump, we firstly investigated its absorption spectrum. By comparing the difference spectra between the strains cR-1 and MY containing control empty plasmids, we quantified the absorption spectrum changes resulting from functional reconstitution of proton pump in the strain cR-1 (Fig. 2a). The absorption spectrum of the recombinant Shewanella cR-1, as depicted in Fig. 2a, exhibits a prominent absorption peak at a wavelength of 566 nm, which corresponds to the characteristic wavelength (557 ± 67 nm) of the natural strain *Haloarcula argentinensis* [30,33]. The absorption spectrum of the recombinant *Shewanella* Arch and Mac, as depicted in Fig. S5a, exhibited a prominent absorption peak at a wavelength of 544 nm and 546 nm, respectively, which corresponded to the characteristic wavelength (566 ± 66 nm) of the natural strain *Halorubrum sodomense* [30,31] and the characteristic wavelength (550 ± 69 nm) of the natural strain *Leptosphaeria maculans* [30,32]. In contrast, the strain MY does not demonstrate any discernible response. Regarding the absorption spectrum detection of proteorhodopsin expressed in *S. oneidensis* [35,36], the expression of *aop3*, *ops, cop-1* in recombinant *S. oneidensis* were preliminarily demonstrated.

#### 3.2.2 Validation of light-driven proton pumping activity in recombinant S. oneidensis

In order to characterize the proton pumping activity of Arch, Mac, cR-1, we employed a high-precision pH meter to monitor the pH variations in the strains Arch, Mac, cR-1 and MY under diverse conditions (Fig. 2b, c and Figs. S5b and c), encompassing the presence or absence of *at*RAL or illumination. The results demonstrated that addition of *at*RAL to the strain cR-1 expressing proton pumps during the illumination phase resulted in a significant decrease in solution pH (∼0.1), indicating efflux of protons by the cells (Fig. 2c). However, upon returning to darkness, there was no further decline in solution pH, which did not revert back to its initial level (Fig. 2c). The aforementioned observations suggest that the recombinant *Shewanella* strain expressing the proton pump cR-1 exhibits active unidirectional transport of protons towards the extracellular environment in response to illumination. When *at*RAL is absent, the solution pH of the strain cR-1 exhibits minor variation (<0.01) under both light and dark conditions (Fig. 2c). In the presence and absence of atRAL, strains Arch and Mac demonstrated similar pH changes to that of strain cR-1, as shown in Figs. S5b and c. In contrast, the solution pH of the strain MY remained almost unaltered irrespective of light exposure or the presence of *at*RAL (<0.01) (as showed in Fig. 2b).

The membrane-permeable weak acid, carbonyl cyanide *m*-chlorophenylhydrazone (CCCP), exhibit high specificity as a protonophore to disrupt the electrochemical proton gradient across the cytoplasmic membrane [37,38]. Following the addition of CCCP to the post-illuminated suspension, both strains MY and cR-1 exhibited transient signs of pH recovery (Fig. 2d). However, the strain MY rapidly restored its pH level almost back to the initial state, whereas the pH of the strain cR-1 did not fully recover to the initial level, while still demonstrated a higher degree of recovery compared to the strain MY (Fig. 2d), thereby emphasizing the significant proton efflux induced by the strain cR-1. Strains Arch and Mac demonstrated similar pH changes to that of strain cR-1 in the presence of CCCP, as shown in Fig. S5d. These results substantiate that the recombinant *Shewanella* expressing the rhodopsins demonstrates functional capability for light-driven proton efflux. Under both dark and light conditions, the voltage output curves of strains MY and cR-1 were presented in Fig. S6. Under illumination, the voltage output of strain cR-1 exhibiting proton efflux was notably higher than that of strain MY. However, the duration of voltage output for strain cR-1 was not only comparable to that of strain MY, but also consistent with the results observed under dark condition for both strains. This indicated that the potential impact of light-driven proton efflux in MFC on the system stability of strain cR-1 during the experimental period was minimal.

As an environmental microorganism capable of metal reduction, the EET function of *S. oneidensis* primarily occurs in the outer and inner membranes, and periplasmic space, which exhibits high sensitivity to fluctuations in extracellular pH levels, rendering *S. oneidensis* an ideal microbial species for studying the impact of PMF on the cellular electrophysiology. In the inner membrane (IM) electron transport chain of the wild-type *S. oneidensis* MR-1, both protons and electrons are transferred to the periplasm through NADH ubiquinone oxidoreductase [39]. Assuming that all these electrons are transported externally via the EET pathway, an equivalent number of protons should be taken into account to ensure overall charge equilibrium throughout the outer membrane (OM). Compared to the strain MY, functional implementation of the rhodopsin cR-1 in the recombinant strain cR-1 facilitates unidirectional proton expulsion, and necessities additional proton expulsion to maintain charge balance of the OM, thereby exhibiting enhanced electron transport during the EET process in MFC.

### 3.3 The recombinant strain cR-1 exhibited light-dependent membrane potential enhancement resulted from proton pumping

**Fig. 3:**
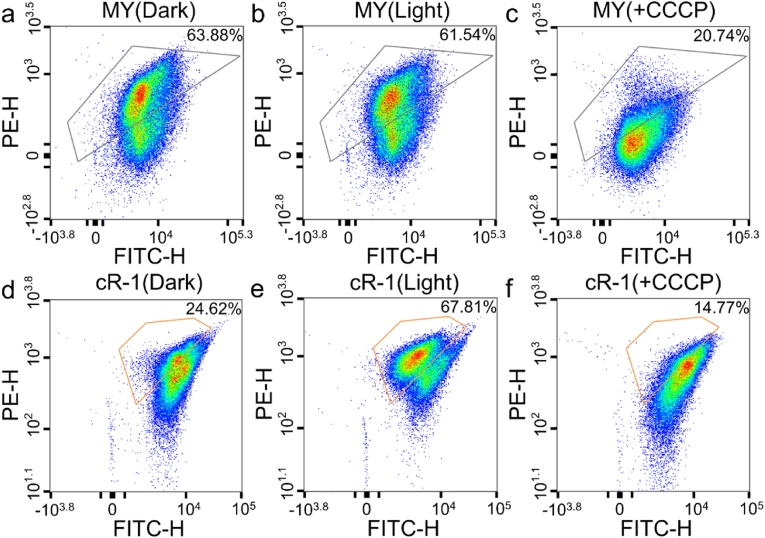
The membrane potential of the strains MY and cR-1 under aerobic culture conditions, quantified in the absence or presence of light, as well as upon addition of 20 μM CCCP using flow cytometer. The cells were incubated with 10 μM atRAL and the membrane potential fluorescence probe DiOC2(3) at a density of 10^6^ cells/mL prior to analysis. The gray box represents the fluorescence detection results of the strain MY under various treatment conditions, while the orange box signifies the fluorescence detection outcomes of the strain cR-1 subjected to diverse treatment conditions. (a) The fluorescence detection results of the strain MY under dark conditions. Gray box: 63.88 %. (b) The fluorescence detection results of the strain MY under light conditions. Gray box: 61.54 %. (c) The fluorescence detection results of the strain MY under 20 μΜ CCCP conditions. Gray box: 20.74 %. (d) The fluorescence detection results of strain under dark conditions. Orange box: 24.62 %. (e) The fluorescence detection results of the strain cR-1 under light conditions. Orange box: 67.81 %. (f) The fluorescence detection results of the strain MY at 20 μΜ CCCP. Orange box: 14.77 %.

Following the confirmation of the rhodopsin gene *cop-1* expression in *S. oneidensis*, we proceeded to investigate the generation of light-induced membrane potentials by monitoring the fluorescence emitted from cells labeled with the lipophilic cyanine dye DioC2(3). The green fluorescence emitted by DiOC_2_(3) is observed within bacterial cells. Upon the addition of *at*RAL and exposure to a specific light source, the strain cR-1 demonstrates enhanced proton pump activity, subsequently leading to an elevation in membrane potential. The enhanced membrane potential induces self-aggregation of the DiOC_2_(3) dye molecules with a red-shifted emission wavelength and increased intensity of the red fluorescence. The results (as depicted in Fig. 3d and e), demonstrate that a significantly higher proportion of the strain cR-1 cells exhibits increased red fluorescence intensity subsequent to the light exposure compared to the dark condition (24.62 %–67.81 %). The cell fluorescence intensity of the strain MY, however, exhibited only a marginal alteration from darkness to light (from 63.88 % to 61.54 %, as shown in Fig. 3a and b), suggesting that its membrane potential remained relatively stable.

A widely employed protonophore, carbonyl cyanide 3-chlorophenyl hydrazone (CCCP), disrupts the membrane potential by abolishing the proton gradient, thereby inducing rapid depolarization and impeding the accumulation of dye within the cytoplasm, ultimately leading to substantial reduction in the red fluorescence intensity. Following the addition of CCCP, both strains MY and cR-1 exhibited a significant reduction in red fluorescence intensity, irrespective of the presence of functional exogenous proton pump (Fig. 3c–f). However, due to its proton efflux leading to an increase in membrane potential, the strain cR-1 experiences a more pronounced disruption in its proton gradient caused by the decrease of CCCP from 61.74 % to 20.74 % in strain MY, and from 67.81 % to 14.77 % in the strain cR-1. These observations further demonstrated the profound effect of the proton translocation across cell membrane on the cell membrane potential.

The simultaneous detection of electrochemical and membrane potential in a single *Shewanella* cell revealed a clear correlation between the EET rate and the membrane potential, thereby emphasizing the feasibility of utilizing membrane potential as a bioenergetic indicator for the EET at the individual cell level [40]. The inner membrane of *Shewanella* undergoes hyperpolarization under anaerobic conditions as a result of proton displacement caused by redox cycling in the quinone pool. This cycle involves reduction of quinones through formate dehydrogenase and lactate dehydrogenase, followed by oxidation of quinols via CymA [41]. In the presence of extracellular electron receptors, electrons are transferred from CymA to these receptors via the Mtr pathway, thereby establishing a connection between EET at the outer membrane (OM) and cation pumping, facilitating transmembrane potential across the inner membrane. Membrane hyperpolarization caused by EET is an important component of proton and sodium dynamics [42,43], and conversely, membrane potential hyperpolarization generated by light-driven proton pumps can also promote EET.

### 3.4 Effect of proton pump expression on ATP level in *S. oneidensis*

#### 3.4.1 Quantification of ATP levels in strains MY and cR-1

In order to verify whether the expression of proton pumps can utilize solar energy to provide energy for recombinant *Shewanella*, we assessed the intracellular ATP levels in the recombinant strain cR-1. Due to the relatively lower proton motive force (PMF) generated by the rhodopsin compared to its own metabolic differentiation [28], nutrient scarcity conditions are generally required for the manifestation of its impact on cellular energy levels. Therefore, we opted to diminish cellular background ATP levels via acetate treatment [44,45], subsequently investigating the augmentative impact of proton pumps on ATP production based on this premise.

**Fig. 4:**
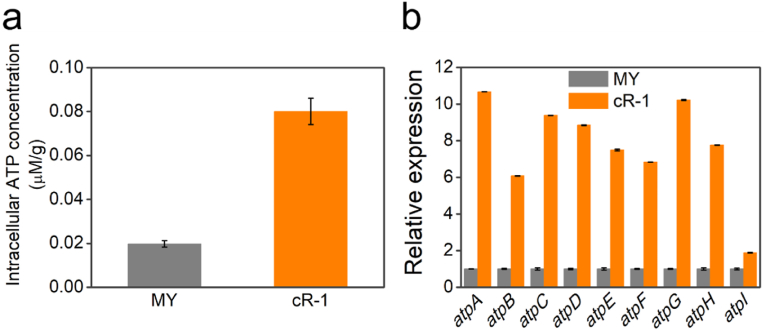
Intracellular ATP measurements and transcriptome analysis of the related genes responsible for ATP synthesis. (a) ATP measurements of the strains MY and cR-1treated with 5 g/L acetate for 4 h. (b) Relative expression levels of the FoF1 ATP synthase-related genes (*atpA-I*) were assessed in the strains MY and cR-1. *atpA*: gene coding FoF1 ATP synthase subunit alpha; *atpB*: gene coding FoF1 ATP synthase subunit A; *atpC*: gene coding FoF1 ATP synthase subunit epsilon; *atpD*: gene coding FoF1 ATP synthase subunit beta; *atpE*: gene coding FoF1 ATP synthase subunit C; *atpF*: gene coding FoF1 ATP synthase subunit B; *atpG*: gene coding FoF1 ATP synthase subunit gamma; *atpH*: gene coding FoF1 ATP synthase subunit delta; *atpI*: gene coding ATP synthase subunit I. The expression level of the aforementioned genes in the strain MY is designated as 1, while the relative expression levels of the above genes in the strains MY and cR-1 are recorded as error bars denote standard error.

Under light condition, the intracellular ATP levels were assessed before and after acetic acid treatment, as depicted in Fig. 4a and Fig. S3, respectively. Prior to acetic acid treatment, the intracellular ATP levels in the strains MY and cR-1 were detected and maintained at a consistent level, as shown in Fig. S3. Following acidic treatment, a significant reduction in intracellular ATP concentration was observed for both strain MY (0.02 ± 0.001 μM/g) and strain cR-1 (0.08 ± 0.006 μM/g), as depicted in Fig. 4a. Under darkness condition, the intracellular ATP levels were assessed before and after acetic acid treatment, as depicted in Figs. S7a and b, respectively. Notably, the experimental group containing the strain cR-1 exhibited considerably higher intracellular ATP levels compared to the control group after acid exposure under light condition. While the strain cR-1 exhibited similar intracellular ATP levels compared to the strain MY after acid exposure under darkness condition. These findings suggest that effective utilization of the proton pump function by the strain cR-1 contributes to the enhanced cellular ATP production.

#### 3.4.2 Analysis of gene expression levels encoding ATP synthetase subunits

Transcriptome analysis revealed that the expression levels of the genes encoding ATP synthase subunits (*atpA-I*) in the strain cR-1 were significantly higher compared to that of the strain MY, with fold changes of 10.68, 6.08, 9.39, 8.85, 7.50, 6.84, 10.22, 7.77 and 1.89, respectively, as shown in Fig. 4b, which was detailed in Table S2. Although *S. oneidensis* has served as a model microorganism for exoelectrogenic studies in the last decades, it remained unclear how ion gradient generated on the cell membrane (such as PMF) during energy production through electron flow influenced electron transport. The process of ATP synthesis is highly active under aerobic conditions, primarily due to the formation of ATP through F-ATPase driven by PMF. However, in anaerobic conditions, fumarate respiration and EET processes do not exhibit comparable levels of activity. Specifically, F-ATPase does not active in the EET-related respiratory activities, resulting in insufficient generation of PMF for ATP synthesis [46,47]. Substrate-level phosphorylation is the primary mechanism for ATP production in anaerobic conditions, while a portion of the ATP pool is also utilized to generate PMF [46]. Hence, when compared to the strain MY, the enhanced intracellular ATP levels observed in the strain cR-1 can be ascribed to the supplementary generation of PMF facilitated by heterologous expression of the proton pump cR-1. Additionally, this conjecture gains further support at molecular level due to the upregulated expression level of the pivotal genes encoding the ATP synthase.

### 3.5 Lactate metabolism analysis

#### 3.5.1 Quantification of lactate metabolism

**Fig. 5:**
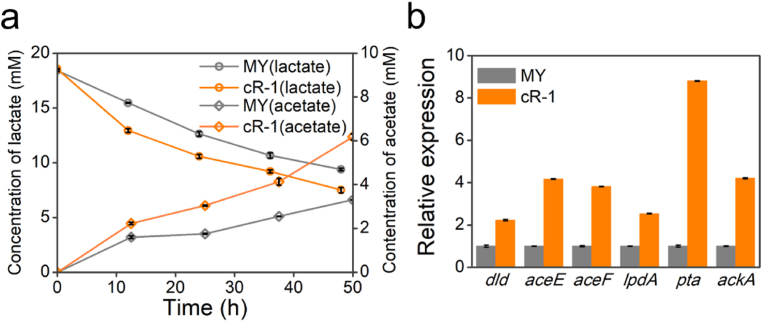
Lactate metabolisms and transcriptome analysis of the related gene. (a) Lactate consumption and acetate accumulation in the MFCs inoculated with the strains MY and cR-1, respectively. MY (lactate): lactate consumption in the MFCs inoculated with the control strain MY; cR-1(lactate): lactate consumption in the MFCs inoculated with the recombinant cR-1; MY (acetate): acetate accumulation in the MFCs inoculated with the control strain MY; cR-1(acetate): acetate accumulation in the MFCs inoculated with the recombinant cR-1. (b) The relative expression levels of lactate metabolisms-related genes were assessed in the strains MY and cR-1. *dld*: gene coding respiratory FAD-dependent d-lactate dehydrogenase; *aceE*: gene coding pyruvate dehydrogenase E1 component; *aceF*: gene coding acetyltransferase component of pyruvate dehydrogenase complex; *lpdA*: gene coding dihydrolipoyl dehydrogenase; *pta*: gene coding phosphate acetyltransferase; *ackA*: gene coding acetate kinase. The expression level of the aforementioned genes in the strain MY is designated as 1, while the relative expression levels of the above genes in the strains MY and cR-1 are recorded as error bars denote standard error.

In MFCs, lactate is oxidized to pyruvate by the respiratory FAD-dependent d-lactate dehydrogenase (Dld). Subsequently, pyruvate undergoes further oxidation to acetyl-CoA by the pyruvate dehydrogenase (PDH) complex (aceE, aceF, and aceG, which identified as dihydrolipoyl dehydrogenase LpdA later) [48]. The conversion of acetyl-CoA to acetate is coupled with ATP production at the substrate level, facilitated by consecutive reactions catalyzed by the phosphate acetyltransferase (Pta) and acetate kinase (AckA) [49]. HPLC detection results for lactate consumption and acetate generation under light and darkness condition were shown in Fig. 5a and Fig. S7c, respectively. Analysis of the concurrently collected MFC samples revealed that strain cR-1 exhibited a more rapid and substantial utilization of lactate compared to the strain MY. Similarly, the strain cR-1 demonstrated enhanced production of acetate, both in terms of rate and quantity, when compared to the strain MY. While under dark condition, the lactate consumption and acetate production of strains cR-1 and MY were comparable to those of strain MY under light condition, as shown in Fig. S7c.

#### 3.5.2 Analysis of gene expression levels related to lactate metabolism

Analysis of the key enzyme genes related to lactate metabolism revealed that the expression level of the gene *dld*, which encodes respiratory FAD-dependent d-lactate dehydrogenase, was 2.48-fold of that in the strain cR-1 than in the strain MY (as illustrated in Fig. 5b). Furthermore, in comparison to the strain MY, the relative expression levels of genes encoding the pyruvate dehydrogenase complex (*aceE*, *aceF*, *lpdA*) exhibit a significant increase in 3.17-fold, 2.82-fold, and 1.53-fold, respectively, in the strain cR-1 than that in the strain MY. The genes encoding phosphate acetyltransferase (*pta*) and acetate kinase (*ackA*) demonstrate a significant increase, with fold changes of 7.80 and 3.21, respectively. The electrochemical chamber of the proton-pump-expressing strain cR-1, cultivated anaerobically on electrodes, demonstrates increased current generation and lactate consumption in the presence of light relative to the strain MY. Considering that the electrons deposited on the electrodes originate exclusively from lactate, it can be inferred that the observed increase in current is directly proportional to the corresponding rise in the lactate consumption. The genes *ackA* and *pta* are indispensable for acetate production and a substantial portion of anaerobic substrate-level ATP production. Compared to the strain MY, the significantly higher expression levels in the strain cR-1 imply that the molecular regulation of lactate metabolism is accountable for the additional production of PMF.

### 3.6 Intercellular NAD (H/^+^) analysis

#### 3.6.1 Quantification of intracellular NAD(H/^+^)

To investigate the impact of the overexpressed proton pump on the intracellular electron pool, the NAD(H/^+^) level was measured in the strains cR-1 and MY. NADH is a crucial electron carrier in cellular metabolism and plays a pivotal role in the respiratory activity of *S. oneidensis* MR-1, serving as an internal electron poor to facilitate EET and enable the generation of exogenous bioelectricity. The genome of *S. oneidensis* MR-1 encodes four NADH dehydrogenases, including Nuo (SO_1009 to SO_1021), Nqr1 (SO_1103 to SO_1108) and Nqr2 (SO_0902 to SO_0907), and Ndh (SO_3517) [42,43,50]. Among these, Nuo is anticipated to pump proton and couple the NADH oxidation with proton translocation, Nqr1 and Nqr2 function as sodium-ion translocators, while Ndh functions as an uncoupling dehydrogenase without ion translocation. Furthermore, both sodium-pumping NADH dehydrogenases (Nqr1 and Nqr2) are ubiquitous in all sequenced genomes within the *Shewanella* genus, whereas the proton-pumping NADH dehydrogenase (Nuo) has only been identified in a limited subset of isolates, including *S. oneidensis* MR-1 [50].

**Fig. 6:**
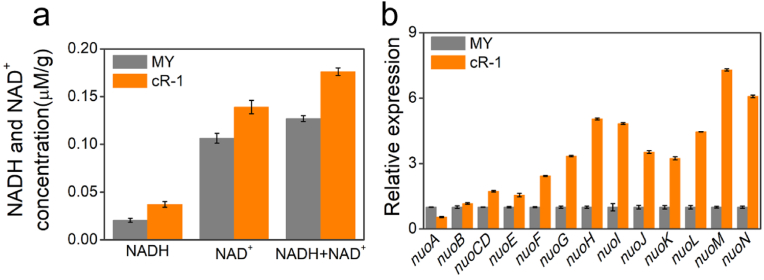
Quantification of intracellular NAD(H/^+^) levels and transcriptome analysis of the related genes. (a) Quantification of the intracellular NADH, NAD^+^, and total NAD(H/^+^) levels of the strains MY and cR-1 in MFCs. (b) Relative expression levels of NADH dehydrogenase-related genes were assessed in the strains MY and cR-1. *nuoA*: gene coding NADH-quinone oxidoreductase subunit A; *nuoB*: gene coding NADH-quinone oxidoreductase subunit B; *nuoCD*: gene coding NADH-quinone oxidoreductase subunit C/D; *nuoE*: gene coding NADH-quinone oxidoreductase subunit E; *nuoF*: gene coding NADH-quinone oxidoreductase subunit F; *nuoG*: gene coding NADH-quinone oxidoreductase subunit G; *nuoH*: gene coding NADH-quinone oxidoreductase subunit H; *nuoI*: gene coding NADH-quinone oxidoreductase subunit I; *nuoJ*: gene coding NADH-quinone oxidoreductase subunit J; *nuoK*: gene coding NADH-quinone oxidoreductase subunitK; *nuoL*: gene coding NADH-quinone oxidoreductase subunit L; *nuoM*: gene coding NADH-quinone oxidoreductase subunit M; *nuoN*: gene coding NADH-quinone oxidoreductase subunit N. The expression level of the aforementioned genes in strain MY is designated as 1, while the relative expression levels of the above genes in the strains MY and cR-1 were recorded as error bars denote standard error.

In this context, we quantified the intracellular NADH (H/^+^) levels in the strains MY and cR-1, and performed a transcriptional analysis of the genes encoding the NADH dehydrogenase Nuo. As depicted in Fig. 6a, under light condition, the strain cR-1 exhibited significantly higher levels of NADH, NAD^+^, and total NAD(H/^+^) compared to the strain MY, with an increase of 50.6 %, 30.7 %, and 38.9 % respectively. The NADH/NAD^+^ ratio of strain cR-1 was 38.4 % higher than that of strain MY. While under dark condition, the levels of NADH, NAD^+^, and total NAD(H/^+^) in strain cR-1 were comparable to those in strain MY, as illustrated in Fig. S7d. Meanwhile, the NADH/NAD^+^ ratio in strain cR-1 was 98.9 % of that observed in strain MY. The elevated intracellular NADH (H/^+^) levels under light condition may be attributed to the heightened intracellular electron production and flow, which is a consequence of increased lactate consumption facilitated by the proton pump.

#### 3.6.2 Analysis of gene expression levels encoding the NADH dehydrogenase nuo

The analysis of related genes encoding Nuo was shown in Fig. 6b. The expression levels of the gene *nuoA-N* in the strain cR-1 were 0.55, 1.17, 1.73, 1.56, 2.43, 3.34, 5.04, 4.83, 3.52, 3.24, 4.46, and 7.30-fold of those in the strain MY. The expression of *nuoA* in the strain cR-1 exhibited a slight decrease compared to that in the strain MY, while the expression of *nuoF-nuoN* was significantly up-regulated, except for *nuoB-nuoE*. Compared to the strain MY, the expression level of the majority of genes encoding NADH-quinone oxidoreductase, specifically *nuoB-nuoN*, were found to be upregulated in the strain cR-1. In comparison to the strain MY, the upregulation of most genes encoding NADH dehydrogenase in the strain cR-1 is consistent with the intracellular NAD(H/^+^) level changes, thereby unveiling the molecular mechanism underlying the augmented intracellular electron pool triggered by heterogeneous expression of proton pumps.

## 4 Conclusion

Three light-driven proton pump genes (*aop3*, *ops*, and *cop-1*) were heterologously expressed in *S. oneidensis,* which were capable of generating remarkably strong outward photocurrent. Among these strains, the recombinant strain cR-1 that expresses the gene *cop-1* exhibited the highest electricity output power density (0.87 ± 0.030 W/m^2^) in MFC, 3.49-fold higher than that of the strain MY (bearing a blank plasmid). We further investigated the expression of the extrinsic light-driven proton pump on the EET efficiency in the strain cR-1 by examining the proton pumping function, energy metabolism, and carbon source utilization. Firstly, the absorption spectrum of the strain cR-1 was examined, revealing a distinct absorption peak at 566 nm, which provided preliminary evidence of the cR-1 expression. Secondly, the comparison of pH changes between the strains cR-1 and MY under light-dark-light conditions revealed that the strain cR-1 exhibited unidirectional proton extrusion in the presence of light, and experienced a collapse in proton gradient upon addition of the protonophore CCCP, further confirming its function as a proton pump. Finally, the membrane potential detection of the strains cR-1 and MY under light and CCCP addition revealed hyperpolarization caused by proton efflux and depolarization induced by CCCP in the strain cR-1. In conclusion, the enhanced EET efficiency of the strain cR-1 was ascribed to the activation of the light-driven proton pump.

The impact of heterologous proton pump on *Shewanella* metabolism and bioenergetics was further assessed by quantifying the ATP production, lactate metabolism, and NAD(H/^+^) generation, which facilitated the elucidation of the intricate mechanism underlying the enhanced EET efficiency facilitated by the heterologous light-driven proton pumps. The intracellular ATP level detection results of the strains MY and cR-1 indicated that the elevated ATP level in the strain cR-1 was attributed to the proton pump-generated PMF. Moreover, the gene expression level of ATP synthase subunit (*atpA-I*) in the strain cR-1 exhibited a significantly higher level compared to that in the strain MY, providing further evidence to substantiate this conclusion. The cR-1 strain exhibited a significant increase in lactate consumption and acetate production compared to the strain MY. The expression levels of the key enzyme genes involved in lactate metabolism were markedly upregulated in the strain cR-1, suggesting that PMF generated by light-driven proton pumping leads to enhanced lactate consumption, resulting in an increased availability of electrons that flow through the EET pathway. The levels of NADH, NAD^+^ and total NAD(H/^+^) in the strain cR-1 were found to be significantly higher than those in the strain MY. Moreover, a majority of the genes encoding the proton-pumping NADH dehydrogenase Nuo exhibited notable upregulation. These findings indicate that the augmentation of the intracellular electron pool by the proton pump leads to enhanced electron transfer from lactate to the EET pathway, thereby amplifying intracellular electron efflux and EET rate.

The prevailing mode of bacterial membrane-dependent energy metabolism is based on the transmembrane circulation of protons. The primary proton pump actively expels H^+^ ions from cytoplasm, generating PMF, which serves as the direct driving force for many essential cellular functions. The EET efficiency was significantly enhanced by heterogeneous expression of light-driven proton pumps, which effectively resolved the predicament of inadequate PMF supply in the course of electron transfer, highlighting that sufficient energy provision is critical in enhancing the EET rate. In addition to enhancing the EET efficiency, the light-driven proton gradient could also be harnessed for driving high-energy synthesis reactions in fine chemicals production, thereby establishing a robust foundation for further advancement in rational engineering of *S. oneidensis* for BESs applications.

## CRediT authorship contribution statement

**Wenqi Ding:** Writing – original draft, Visualization, Validation, Methodology, Investigation, Formal analysis, Data curation, Conceptualization. **Tong Lin:** Writing – original draft, Visualization, Validation, Methodology, Investigation, Formal analysis, Data curation, Conceptualization. **Yun Yang:** Validation, Methodology, Investigation, Formal analysis, Conceptualization. **Wen-Wei Li:** Validation, Supervision, Project administration, Conceptualization. **Shaoan Cheng:** Validation, Supervision, Project administration, Conceptualization. **Hao Song:** Writing – review & editing, Supervision, Resources, Project administration, Methodology, Funding acquisition.

## Declaration of competing interest

The authors declare that they have no known competing financial interests or personal relationships that could have appeared to influence the work reported in this paper.
