# Supplementary material for: Proton motive force generated by microbial rhodopsin promotes extracellular electron transfer
Source: Synth Syst Biotechnol. 2025 Jan 7;10(2):410–20. doi: 10.1016/j.synbio.2025.01.001 (PMC11786069; doi:10.1016/j.synbio.2025.01.001)
Supplement: Multimedia component 1 [file mmc1.docx]

**Supporting Information**

**Proton motive force generated by microbial rhodopsin promotes extracellular electron transfer**

Wenqi Ding^a^ **^‡^**, Tong Lin^b^ **^‡^**, Yun Yang^c^, Wen-Wei Li^d^, Shaoan Cheng^e^, and Hao Song^a,f^ *

^a^ Frontiers Science Centre for Synthetic Biology (Ministry of Education), and Key Laboratory of Systems Bioengineering, School of Chemical Engineering and Technology, Tianjin University, Tianjin 300072, China

^b^ College of Life Science, Langfang Normal University, Langfang, Hebei 065000, China

^c^ Beijing Advanced Innovation Centre for Biomedical Engineering, Key Laboratory for Biomechanics and Mechanobiology of Ministry of Education, School of Engineering Medicine, Beihang University, Beijing 100083, China

^d^ Chinese Academy of Sciences Key Laboratory of Urban Pollutant Conversion, Department of Environmental Science and Engineering, University of Science & Technology of China, Hefei 230026, China

^e^ State Key Laboratory of Clean Energy, Department of Energy Engineering, Zhejiang University, Hangzhou, 310027, China

^f^ Haihe Laboratory of Sustainable Chemical Transformations, Tianjin, 300192, China

^‡^ These authors contribute equally to this work.

* Corresponding authors.

E-mail: [hsong@tju.edu.cn](mailto:hsong@tju.edu.cn) (H.S.)


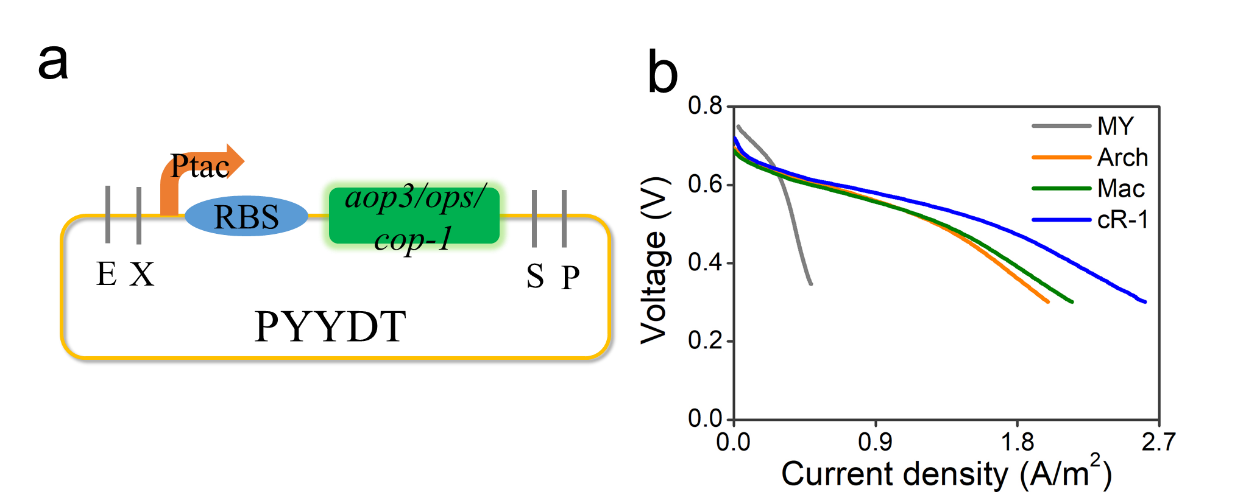


**Figure S1.** Expression of microbial rhodopsins *aop3/ops/cop-1* in the recombinant *Shewanella oneidensis* strains Arch, Mac and cR-1. (a) Plasmid map of the PYYDT vector expressing the gene *aop3, ops,* or *cop-1* under the control of the inducible promoter P_tac_ in strains Arch, Mac and cR-1, respectively. E, X, S, P designate the restriction enzyme sites of EcoRI, XbaI, SpeI, PstI, respectively. (b) MFC polarization discharge curves obtained by linear sweep voltammetry (LSV) with a scan rate of 0.1 mV/s.


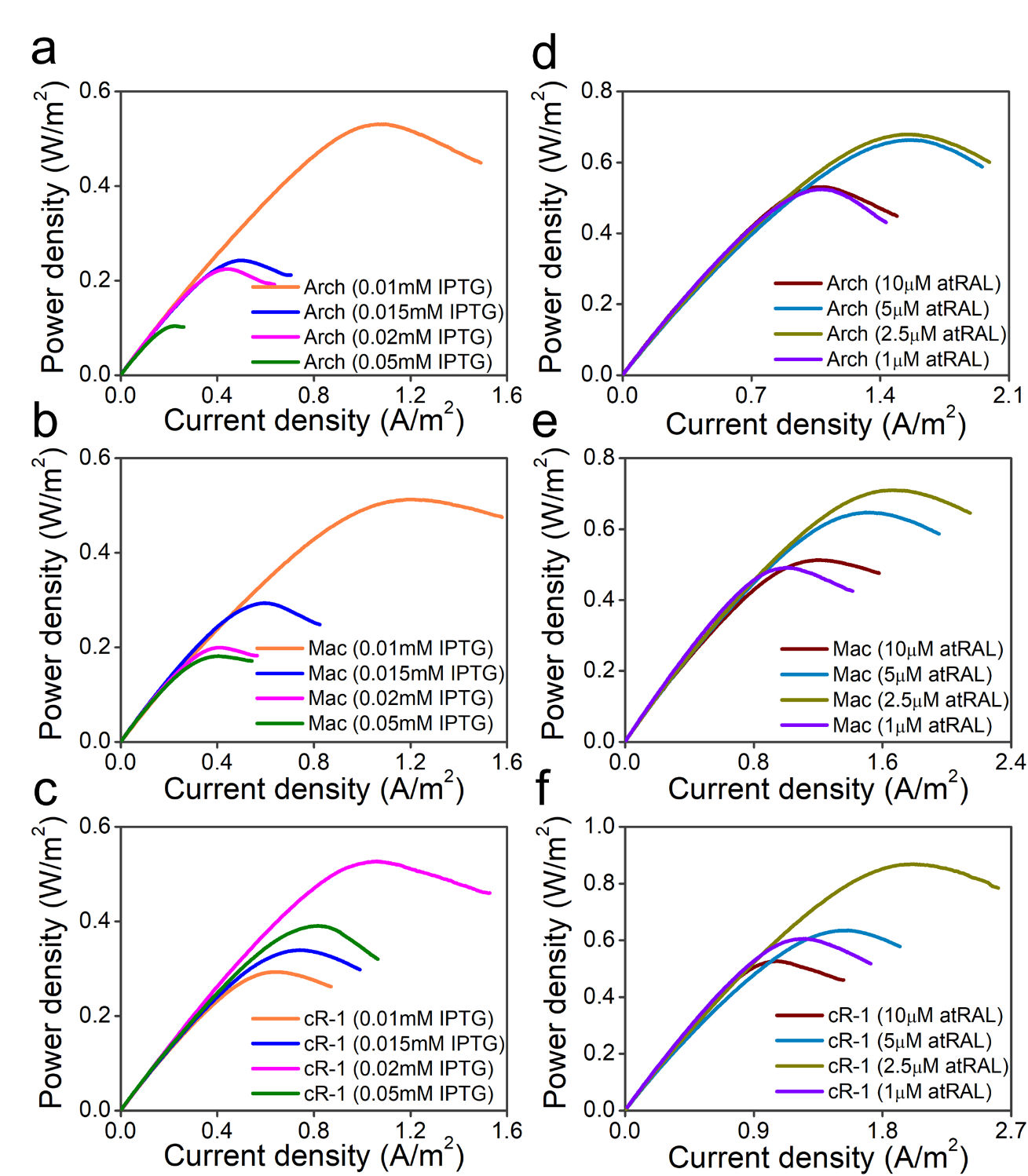


**Figure S2.** Power generation of the recombinant strains Arch, Mac and cR-1 with different concentrations of IPTG or *at*RALin MFCs. The MFCs’ power density output curves obtained by linear sweep voltammetry (LSV) with a scan rate of 0.1 mV/s. (a) Power generation of the strain Arch with different concentrations of IPTG added by 10μΜ *at*RAL. (b) Power generation of the strain Mac with different concentrations of IPTG added by 10μΜ *at*RAL. (c) Power generation of the strain cR-1 with different concentrations of IPTG added by 10μΜ *at*RAL. (d) Power generation of the strain Arch with different concentrations of *at*RAL induced by 0.01mM IPTG. (e) Power generation of the strain Mac with different concentrations of *at*RAL induced by 0.01mM IPTG. (f) Power generation of the strain cR-1 with different concentrations of *at*RAL induced by 0.02mM IPTG. Data are the average of three replicates for each strain.


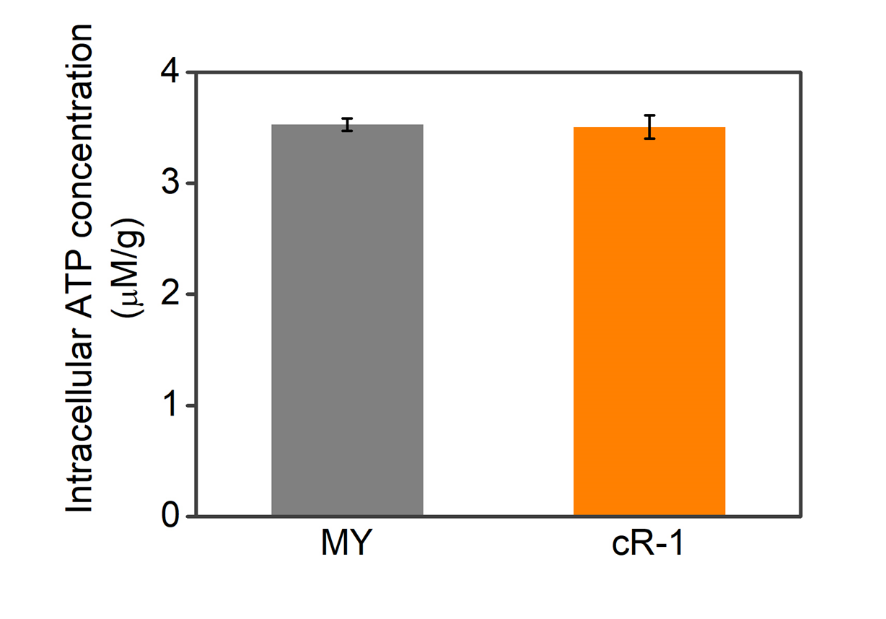


**Figure S3**. Quantification of the intracellular ATP concentrations of the strains MY and cR-1 before the acetic acid treatment.


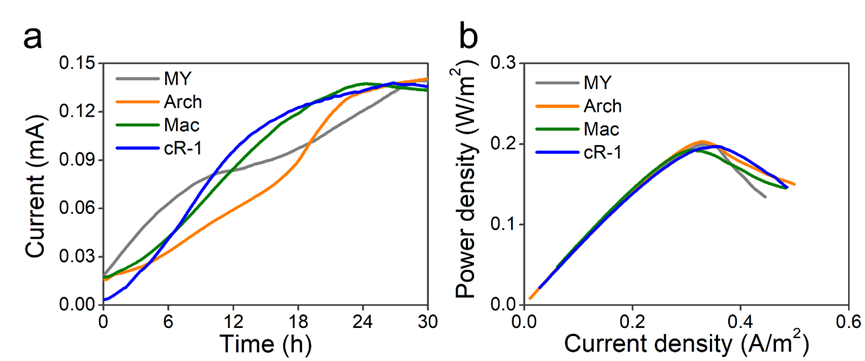


**Figure S4.** Electrochemical analysis of the strain MY (bearing an empty plasmid) and three recombinant strains Arch, Mac, cR-1 under darkness condition. (a) Chronoamperometry of the strains MY, Arch, Mac, and cR-1 interacting with working electrode (carbon cloth) poised at 0.2 V *vs.* Ag/AgCl under darkness condition. (b) Power density output curves of the strains MY, Arch, Mac, and cR-1 under darkness condition. Data are the average of three replicates for each strain.


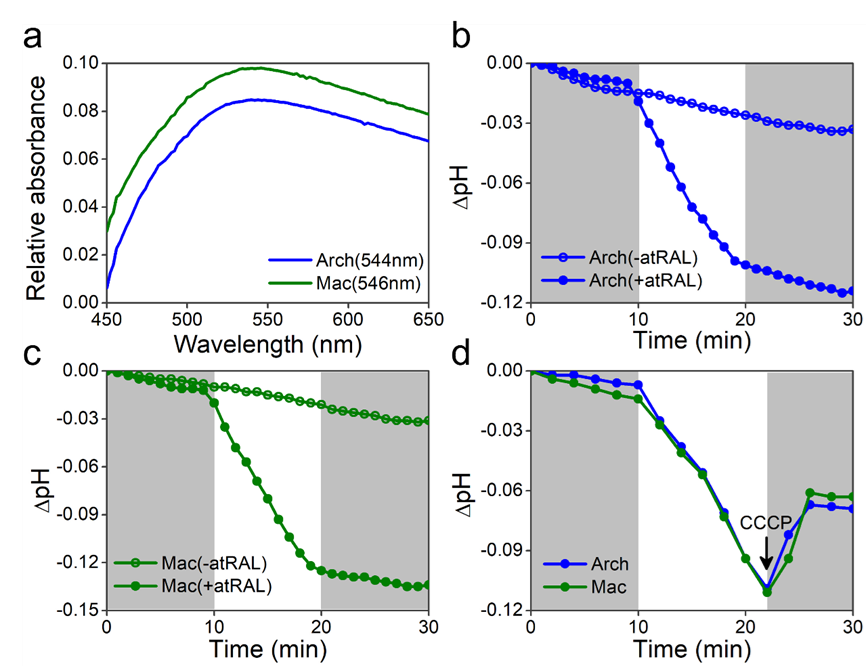


**Figure S5.** Absorption spectrum and validation of the light-driven proton pumping activity of the recombinant strains Arch and Mac. Darkness condition (0-10 min and 20-30 min) are represented by gray shade, while the light condition (10-20 min) is represented by white shade. (a) Absorption spectrum of strains Arch and Mac. Relative absorbance is the absorbance change of crude membrane after adding *at*RAL. (b) The pH changes of strain Arch were observed under a dark-light-dark cycle, both in the absence (blue hollow: -*at*RAL) and presence (blue solid: +*at*RAL) of *at*RAL. (c) The pH changes of strain Mac were observed under a dark-light-dark cycle, both in the absence (green hollow: -*at*RAL) and presence (green solid: +*at*RAL) of *at*RAL. (d)The pH changes of strains Arch and Mac were observed after addition of 20 μM CCCP following10 min of light exposure. The black arrow represents the addition of CCCP (20 μM).


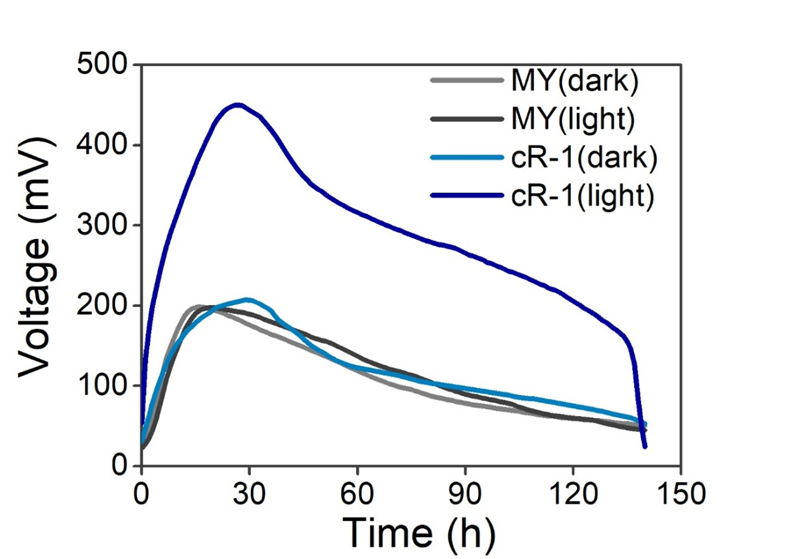


**Figure S6**. The voltage output curve of MFCs inoculated with strains MY and cR-1 under light and darkness conditions. Data are the average of three replicates for the strains.


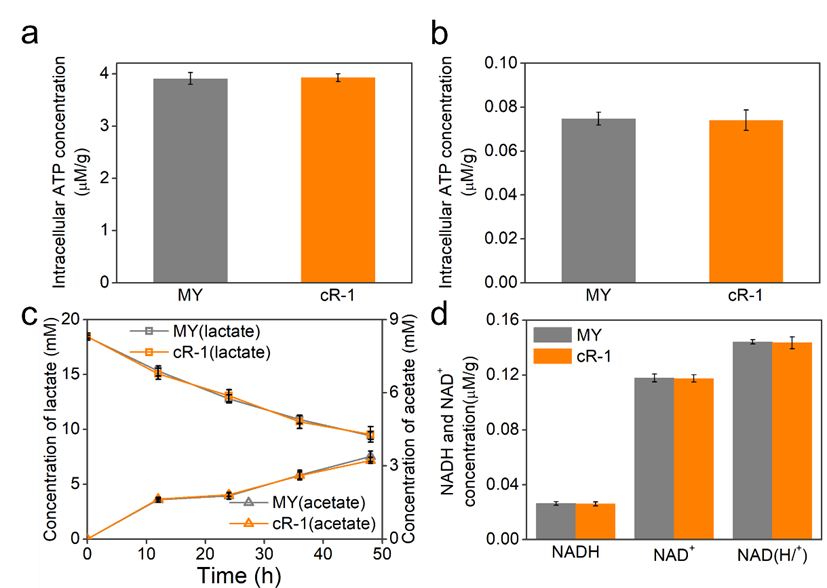


**Figure S7**. Measurements of intracellular ATP, NAD(H/^+^) levels and lactate metabolisms. (a) Quantification of the intracellular ATP concentrations of strains MY and cR-1 before the acetic acid treatment under darkness condition. (b) Quantification of the intracellular ATP concentrations of strains MY and cR-1 treated with 5 g/L acetate for 4 hours under darkness condition. (c) Lactate consumption and acetate accumulation in the MFCs inoculated with strains MY and cR-1 under darkness condition, respectively. MY (lactate): lactate consumption in the MFCs inoculated with the control strain MY; cR-1(lactate): lactate consumption in the MFCs inoculated with the recombinant cR-1; MY (acetate): acetate accumulation in the MFCs inoculated with the control strain MY; cR-1(acetate): acetate accumulation in the MFCs inoculated with the recombinant cR-1. (d) Quantification of the intracellular NADH, NAD^+^, and total NAD(H/^+^) levels of the strains MY and cR-1 in MFCs under darkness condition.

**Table S1.** The codon-optimized sequences of the genes *aop3*, *ops*, and *cop-1*

| *aop3* 777bp |
| --- |
| atggatccaatcgctttacaagctggttacgatttattaggtgatggtcgtccagaaactttatggttaggtatcggtactttattaatgttaatcggtactttctacttcttagttcgtggttggggtgttactgataaagatgctcgtgaatactacgctgttactatcttagttccaggtatcgcttctgctgcttacttatctatgttcttcggtatcggtttaactgaagttactgttggtggtgaaatgttagatatctactacgctcgttacgctgattggttattcactactccattattattattagatttagctttattagctaaagttgatcgtgttactatcggtactttagttggtgttgatgctttaatgatcgttactggtttaatcggtgctttatctcacactgctatcgctcgttactcttggtggttattctctactatctgtatgatcgttgttttatacttcttagctacttctttacgttctgctgctaaagaacgtggtccagaagttgcgtcgacattcaacactttaactgctttagttttagttttatggactgcttacccaatcttatggatcatcggtactgaaggtgctggtgttgttggtttaggtatcgaaactttattattcatggttttagatgttactgctaaagttggtttcggtttcatcttattacgttctcgtgctatcttaggtgatactgaagctccagaaccaagtgctggtgcggatgtaagcgctgctgattaa |
| *ops* 942bp |
| atgatcgttgatcaattcgaagaagttttaatgaaaacttctcaattattcccattaccaactgctactcaatctgctcaaccaactcacgttgctcccgttccaactgtcctaccagatactccaatctacgaaactgttggtgattctggttctaaaactttatgggttgttttcgttttaatgttaatcgcttctgctgctttcactgctttatcttggaaaatcccagttaaccgtcgtttataccacgttatcactactatcatcactttaactgctgctttatcttacttcgctatggctactggtcacggtgttgctttaaacaaaatcgttatccgtactcaacacgatcacgttccagatacttacgaaactgtttaccgtcaagtttactacgctcgttacatcgattgggctatcactactccattattattattagatttaggtttattagctggtatgtctggtgctcacatcttcatggctatcgttgctgatttaatcatggttttaactggtttattcgctgctttcggttctgaaggtactccacaaaaatggggttggtacactatcgcttgtatcgcttacatcttcgttgtttggcacttagttttaaacggtggtgctaacgctcgtgttaaaggtgaaaaattacgttctttcttcgttgctatcggtgcttacactttaatcttatggactgcttaccccatcgtatggggtctcgctgatggtgcgcgtaaaatcggtgttgatggtgaaatcatagcttacgctgtactagatgttttagctaaaggtgttttcggtgcttggttattagttactcacgctaacttacgtgaatctgatgttgaattaaacggtttctgggctaacggtttaaaccgtgaaggtgctatccgtatcggtgaagatgatggtgcttaa |
| *cop-1* 753bp |
| atgccagaaccaggttctgaagctatctggttatggttaggtactgctggtatgttcttaggtatgttatacttcatcgctcgtggttggggtgaaactgattctcgtcgtcaaaaattctacatcgctactatcttaatcactgctatcgctttcgttaactacttagctatggctttaggtttcggtttaactatcgttgaatttgctggtgaagaacacccaatctactgggctcgttactctgattggttattcactactccattattattatacgatttaggtttattagctggtgctgatcgtaacactatcacttctttagtttctttagatgttttaatgatcggtactggtttagttgctactttatctccaggtagtggcgtgctatctgctggtgctgaacgtttagtttggtggggtatctcgactgcgttcttactcgttttattatacttcttattctcttctttatctggtcgtgttgctgatttaccatctgatactcgttctactttcaaaactttacgtaacttagtaactgttgtgtggctcgtttacccagtatggtggctcatcggtactgagggcatcggcttagttggtatcggcatcgaaactgctggtttcatggttatcgatttaactgctaaagttggtttcggtatcatcttattacgttctcacggtgttttagatggtgctgctgaaactactggtactggtgctactccagctgatgattaa |

**Table S2.** The genes studied in this work

| Gene | Gene name | Fold change of the abundance in cR-1 vs. MY | p-value | Description |
| --- | --- | --- | --- | --- |
| SO_4749 | *atpA* | 3.42 | 0 | F0F1 ATP synthase subunit alpha |
| SO_4753 | *atpB* | 2.60 | 0 | F0F1 ATP synthase subunit A |
| SO_4746 | *atpC* | 3.23 | 0 | F0F1 ATP synthase subunit epsilon |
| SO_4747 | *atpD* | 3.15 | 0 | F0F1 ATP synthase subunit beta |
| SO_4752 | *atpE* | 2.91 | 0 | F0F1 ATP synthase subunit C |
| SO_4751 | *atpF* | 2.77 | 0 | F0F1 ATP synthase subunit B |
| SO_4748 | *atpG* | 3.35 | 0 | F0F1 ATP synthase subunit gamma |
| SO_4750 | *atpH* | 2.96 | 0 | FOF1 ATP synthase subunit delta |
| SO_1521 | *dld* | 1.16 | 2.92E-70 | FAD-binding oxidoreductase |
| SO_0424 | *aceE* | 2.06 | 0 | pyruvate dehydrogenase |
| SO_0425 | *aceF* | 1.93 | 0 | pyruvate dehydrogenase complex dihydrolipoyllysine-residue acetyltransferase |
| SO_0426 | *lpdA* | 1.34 | 0 | dihydrolipoyl dehydrogenase |
| SO_2916 | *pta* | 3.14 | 0 | phosphate acetyltransferase |
| SO_2915 | *ackA* | 2.07 | 0 | acetate kinase |
| SO_1021 | *nuoA* | -0.87 | 1.89E-33 | NADH-quinone oxidoreductase subunit A |
| SO_1020 | *nuoB* | 0.22 | 7.17E-05 | NADH-quinone oxidoreductase subunit B |
| SO_1019 | *nuoCD* | 0.79 | 3.25E-66 | NADH-quinone oxidoreductase subunit C/D |
| SO_1018 | *nuoE* | 0.64 | 4.66E-13 | NADH-quinone oxidoreductase subunit NuoE |
| SO_1017 | *nuoF* | 1.28 | 1.25E-134 | NADH-quinone oxidoreductase subunit NuoF |
| SO_1016 | *nuoG* | 1.74 | 0 | NADH-quinone oxidoreductase subunit NuoG |
| SO_1015 | *nuoH* | 2.34 | 6.01E-175 | NADH-quinone oxidoreductase subunit NuoH |
| SO_1014 | *nuoI* | 2.26 | 6.24E-104 | NADH-quinone oxidoreductase subunit NuoI |
| SO_1013 | *nuoJ* | 1.81 | 1.14E-97 | NADH-quinone oxidoreductase subunit J |
| SO_1012 | *nuoK* | 1.69 | 8.05E-43 | NADH-quinone oxidoreductase subunit NuoK |
| SO_1011 | *nuoL* | 2.16 | 0 | NADH-quinone oxidoreductase subunit L |
| SO_1010 | *nuoM* | 2.87 | 0 | NADH-quinone oxidoreductase subunit M |
| SO_1009 | *nuoN* | 2.61 | 0 | NADH-quinone oxidoreductase subunit NuoN |
